# Understanding the brain mechanisms behind anxiety disorders in late life: progress and limitations of the past 15 years

**DOI:** 10.3389/fpsyt.2026.1797635

**Published:** 2026-08-19

**Authors:** Attakias T. Mertens, Gaelle E. Doucet

**Affiliations:** 1 Institute for Human Neuroscience, NE, United States; 2 Center for Pediatric Brain Health, NE, United States; 3 Department of Pharmacology and Neuroscience, Creighton University School of Medicine Omaha, NE, United States

**Keywords:** amygdala, functional MRI, generalized anxiety disorder, late-life anxiety, structural MRI

## 1 Introduction

The number of individuals aged 60 years and older will reach a new global high in 2030 and is projected to continue increasing thereafter (1). With this, research on healthcare, age-related disorders, and mental health in aging has increased. Anxiety disorders are among the most common mental health disorders worldwide (2). Accumulating evidence has further suggested that the presentation and symptoms of anxiety likely differ across the lifespan and by sex (3, 4). While rates of most anxiety disorders are reported to decrease with age, rates of generalized anxiety disorder (GAD) have similar rates in both younger and older adults (5). Roughly half of the GAD cases reported in older adults have an onset after 50 years of age (5) and show mixed results on whether prevalence differs by sex (6, 7). Given this, we will focus on GAD within the context of later life stages for this opinion and include some discussion of sex effects when relevant.

The presence of clinically relevant anxiety symptoms in older adults has been estimated to vary between 15% and 56% within medical settings (8). Additionally, a recent meta-analysis on studies that implemented anxiety measures specific to geriatric populations (the Geriatric Anxiety Scale and Geriatric Anxiety Inventory), estimated that roughly 25% of older adults experience some degree of anxiety symptoms (4). Anxiety symptoms in older age have a number of comorbidities including cognitive decline, poorer quality of life, cardiovascular issues, and other mental health issues, especially depression (5, 6, 9–11). With this, there is a pressing need to better understand the neurological mechanisms behind anxiety disorders at each stage of life. Given that age significantly impacts many other brain systems such as those responsible for executive functioning (12) and emotional regulation (13), it is imperative to also better understand the impact of age on the mechanisms linked to high anxiety levels as they may differ throughout the lifespan (see our opinion on that topic in the next section). Neuroimaging techniques provide noninvasive insights into such neural structure and function. In the last 15 years, the number of magnetic resonance imaging (MRI) studies has exploded with a progressive improvement in signal to noise (SNR) ratio, including better spatial and temporal resolutions, which has led to more reliable findings. However, due to the high number of publications, challenges remain in regard to the reproducibility of these neuroimaging findings, and one should remain critical until results can be robustly reproduced, regardless of sequences, head motion, or scanners. Due to the prevalence of neuroimaging research using MRI, we chose to focus on structural and functional (fMRI) MRI findings. In this context, for this special research topic celebrating the 15^th^ anniversary of Frontiers in Psychiatry, we summarized different and specific aspects of MRI literature from the last 15 years, related to anxiety with a focus on late stages of life. We aim to highlight both the major discoveries made as much as the major challenges that remain to be further investigated. Academic search engines were used to find articles with the following inclusion criteria: must implement MRI measures, be published in the specified timeframe, and investigate some form of anxiety/late-life GAD. While this is far from an exhaustive review, we hope that this opinion will be an eye-opener that provides some guidance and suggestions for the future research of the next 15 years.

## 2 Brain structure linked with anxiety: findings from structural MRI studies

Already 10 years ago, Andreescu and Varon (14) noted a lack of neuroimaging research for late-life anxiety/GAD compared to younger populations, particularly for structural imaging. This was further highlighted by a review from Kolesar et al. (15) that covered brain structural and functional abnormalities in individuals with GAD, regardless of age. This review noted overall structural neural abnormalities in individuals with GAD, including smaller hypothalamic (16) and hippocampal gray matter volumes (GMV; 17, 18), larger amygdala GMV (19), and larger white matter volume in the dorsolateral prefrontal cortex (dlPFC; 18). However, of the 85 studies included in the review, only eight had older adult participants and only two of these reported structural differences associated with GAD. Both studies included small sample sizes and did not investigate for sex-related effects. The first of these studies, from Mohlman et al. (20), found that the volume of the medial orbitofrontal cortex (OFC) had a positive association with worry in those with GAD, but not in asymptomatic peers within a sample of 30 older adults aged 60 to 77. The second structural study by Andreescu et al. (21) included a sample of 59 older adults and found that those with GAD had higher mean diffusivity (MD) in the white matter of the left pallidum and lower MD in the left middle fronto-orbital gyrus. In addition to this, those with GAD had less cortical thickness in the medial OFC and left rostral ACC, and smaller volume of the right inferior frontal gyrus. Unfortunately, none of the results in this study survived corrections for multiple comparisons. With this, moderate to large effect sizes (ranging from 0.54 to 1.06) were noted from the results, leading the researchers to suggest that a larger sample might be able to confirm these results.

The Enhancing Neuroimaging Genetics through Meta-Analysis (ENIGMA) collaboration seeks to ameliorate the issue of small sample sizes through worldwide, multi-site collaboration projects focused on a common topic (22). One such collaboration within ENIGMA includes the Anxiety Working Group, committed to collecting structural MRI data in those with and without anxiety disorders (23, 24). A recent study, including data from 28 research sites, reported virtually no statistical differences across structural brain metrics between individuals with and without GAD (25). Furthermore, null results were also reported for GAD-related sex effects. All findings were based on both regional and vertex-wise metrics related to cortical thickness, cortical surface area, and subcortical volume. The results showed small effect sizes (≤0.005; 25) from a sample that included over 4,000 individuals aged 5 to 90 years old. These results contrast with previous studies, including those within older adults that we have discussed. While this implies that sample size may not be the main cause for the lack of consistent findings across studies, there are key points to consider. Primarily, it should be noted that, although a majority of the lifespan was represented in the ENIGMA Anxiety study (5–90 years), individuals within different age ranges were severely unbalanced (25). Mainly, with relevance to this opinion, of the over 4,000 individuals included, less than 100 were between the ages of 60 and 90 years. Thus, given their underrepresentation even in a large dataset, there is still motivation for future studies to further investigate potential neural structural differences related to GAD within the aging population specifically. Assessing for linear effects of age on brain structure associated with GAD across the lifespan implies the notion that GAD has uniform, compounding impacts across brain regions. As discussed earlier, there are notable differences in symptom expression and sources of worry across age. This indicates the potential for differential neural changes associated with GAD depending on age, which are likely further compounded by disease duration. The latter introduces a final potential explanation for the lack of findings in Harrewijn et al. (25), as the study included those with current and lifetime GAD in the same analyses. While the models were rerun for only those with current GAD, from which no significant results were found, this does not address the need for comparison across age groups. Therefore, we believe that a good direction forward would be to assess neural changes within specific age groups in conjunction with assessments that are able to detect differential anxiety expressions. Additionally, longitudinal collection of such data could aid in a better understanding of how anxiety changes throughout the lifespan.

## 3 Brain functions related to anxiety: findings from functional MRI studies

Of the MRI studies that focused on anxiety in older age, more have implemented fMRI than structural MRI. These studies have primarily found differences in functional activation and functional connectivity (FC) measures from task and/or resting-state fMRI. A meta-analysis of 367 task-based fMRI studies comparing those with mood and anxiety disorders (MAD) to non-symptomatic young adults (any studies focused on older populations were excluded), noted differences in neural activity across six common regions (26). Of these regions, individuals with MAD showed reduced activation across tasks in three, including the inferior prefrontal cortex/insula, inferior parietal lobule, and putamen, implicating poorer salience and inhibitory processing. The remaining three regions, the left amygdala/parahippocampal gyrus, left thalamus, and the perigenual/dorsal anterior cingulate cortex (dACC), were more inconsistent in their activation pattern across tasks, but primarily showed higher level of activation, compared to healthy individuals. Interestingly, the amygdala, ACC, and insula have been shown to be associated with anxiety and worry in late life. All these findings come from four task-based fMRI studies, which all used either a similar worry induction and reappraisal task (27–29) or an emotion regulation task (30). These studies displayed differences in activation and FC related to these regions, among others, in older adults with and without GAD. Additionally, all these studies either did not assess sex differences (27, 28) or controlled for them (29, 30). Altogether, these results are purely within the context of the specific task used and come from smaller to moderate sample sizes ranging from 17 (27) to 59 (28) for participants with or without GAD specifically, and 131 (29) participants with or without an anxiety/depressive disorder. While these studies provide an excellent starting point, it should be noted that most of the findings available come from one group. With this, there is a clear need for additional, independent researchers to help validate current results in separate, larger samples and further contribute to the literature within other cognitive contexts in younger and older adults with GAD.

## 4 Conclusion

**Figure 1:**
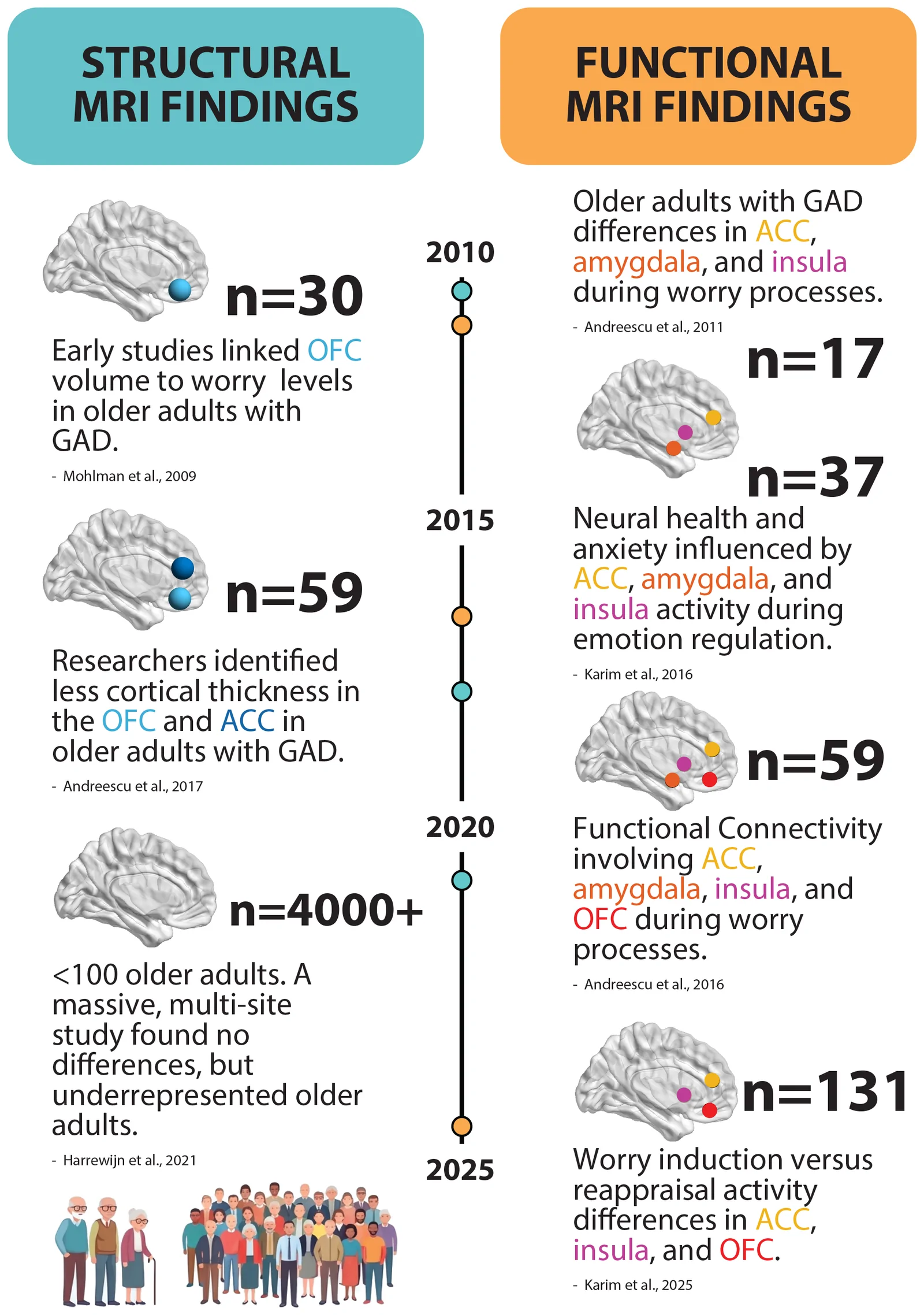
Summary visual of the major findings showing common regions involved in late-life anxiety from the past 15 years of MRI research. Studies are grouped as they are discussed in the opinion, by structural MRI (left, cool colors) and functional MRI (right, warm colors). A timeline separates the two groups, with colored dots indicating the year of publication, relative to the depicted studies. Region colors in text are matched to those in the images. ACC, anterior cingulate cortex. GAD, generalized anxiety disorder. OFC, orbitofrontal cortex.

Across structural and functional MRI research, some brain regions do seem to play a part in anxiety/worry in older age. Two regions specifically were found across methodologies, including the OFC and ACC (**Figure 1**). The findings involving these regions, however, come from few studies that included small samples, making it difficult to place full confidence in their results. Additionally, these studies lack proper investigation of sex effects, which could present further differences in those with GAD. For structural work, large dataset findings have not provided more evidence specific to older adults and large dataset findings for functional work are currently lacking. Therefore, future research is needed to further investigate and validate the potential underlying neural mechanisms behind GAD across the life stages, particularly in late adulthood, and if sex further impacts them. As such, it is important for future work to consider changes associated with GAD that are specific to older adults. Thus, careful consideration will be necessary for the assessments used going forward. More measurements should be implemented that are flexible to stressors, symptoms, and behaviors related to an individual’s age, without excluding those that may occur in other ages. Such studies will be necessary for understanding the neural mechanisms involved in GAD during late life. Additionally, longitudinal samples including both those with life-long and late onset GAD will be imperative to determine the impact of disease duration and if different neural alterations occur. Regarding MRI work, while larger sample sizes are a common goal in research, the overall lack in the number of studies focused on GAD in older age is a concern. Both large structural and functional research studies are currently lacking. Therefore, while the currently available publications have created a starting point, there is still much left unknown to build upon.
